# Integrated Multi‐Omics Analysis and Experimental Validation Identify UBE2Q2 as a Candidate Molecule Target in Intervertebral Disc Degeneration

**DOI:** 10.1111/jcmm.71292

**Published:** 2026-07-18

**Authors:** Wenqiang Cheng, Jianye Yang, Fangfang Liu, Meijun Chen, Hao Pan, Dong Wang, Weihui Qi, Jintao Hu

**Affiliations:** ^1^ Department of Orthopaedics Hangzhou Traditional Chinese Medicine Hospital Affiliated to Zhejiang Chinese Medical University Hangzhou China; ^2^ Department of Orthopaedics Hangzhou Dingqiao Hospital Hangzhou China

**Keywords:** bioinformatics, intervertebral disc degeneration, single‐cell RNA sequencing, UBE2Q2, ubiquitin‐conjugating enzyme, weighted gene co‐expression network analysis

## Abstract

Intervertebral disc degeneration (IVDD) is a primary cause of low back pain, with its molecular mechanisms remaining incompletely understood. Ubiquitin‐conjugating enzymes (E2 enzymes) play critical roles in protein degradation and maintenance of cellular homeostasis, yet their expression profiles and functional significance in IVDD remain largely unexplored. Using Gene Expression Omnibus (GEO) datasets (GSE70362 and GSE23130), we performed differential expression analysis, intersected with ubiquitination‐related genes, and applied weighted gene co‐expression network analysis (WGCNA) to identify key E2 enzymes in IVDD. Single‐cell RNA sequencing (GSE251686) characterized UBE2Q2 expression in intervertebral disc cell subpopulations. qRT‐PCR, Western blot, immunohistochemistry, and immunofluorescence validated UBE2Q2 expression in human degenerated nucleus pulposus (NP) tissues and in vitro rat NP cell degeneration models. Bioinformatics analyses showed significant UBE2Q2 upregulation in IVDD, with WGCNA identifying it as a hub gene closely associated with degeneration. Consistently, single‐cell data confirmed predominant UBE2Q2 expression in NP cells, with markedly higher levels in IVDD (*p* < 0.05). Moreover, experimental validation consistently demonstrated elevated UBE2Q2 mRNA and protein in degenerated human NP tissues and rat models, with stronger immunohistochemical and immunofluorescent signals in degenerated samples (*p* < 0.05). This study represents the first identification of UBE2Q2 as a potential biomarker and a new molecular target for IVDD, thereby providing a foundation for future functional and mechanistic investigations.

## 1 Introduction

Intervertebral disc degeneration is the leading cause of low back pain (LBP) and imposes a major global epidemiological burden. According to the 2021 Global Burden of Disease (GBD) study, approximately 619 million people worldwide suffered from LBP in 2020, with projections indicating an increase to 843 million by 2050 [1]. LBP is highly prevalent among middle‐aged and older adults and leads to considerable healthcare expenditure, lost productivity, and socioeconomic burden [2, 3]. As the primary pathological substrate of LBP, IVDD markedly impairs patients' quality of life [4, 5]. Current treatments for intervertebral disc degeneration mainly rely on conservative therapies and surgical interventions. However, these approaches primarily provide symptomatic relief and fail to reverse the degenerative process or restore the biological function of the intervertebral disc. Moreover, they are frequently associated with complications, including recurrence and adjacent segment degeneration [6, 7]. Therefore, identifying key molecular players in IVDD and evaluating their potential as biomarkers is of considerable clinical significance.

IVDD pathogenesis involves multiple interconnected processes, including extracellular matrix (ECM) degradation, inflammation, and cellular homeostatic imbalance [8, 9]. Previous research has shown that the ubiquitin‐proteasome system (UPS) is critical for maintaining proteostasis [10, 11]. In recent years, the involvement of UPS in IVDD has garnered growing interest. Dysregulation of UPS has been shown to aggravate oxidative stress and inflammation. For instance, the deubiquitinase USP11 stabilizes Sirt3 to suppress ferroptosis and thereby mitigates IVDD [12]. Likewise, MARCHF8‐mediated TGFBI ubiquitination activates NF‐κB signalling, which promotes nucleus pulposus cell apoptosis, ECM degradation, and inflammation [13]. These observations suggest that UPS constitutes a key regulatory hub in IVDD. In the UPS enzymatic cascade, the E2 ubiquitin‐conjugating enzyme family performs a central function. These E2 enzymes serve as intermediates in ubiquitination, working with E1‐activating enzymes and E3 ligases to transfer activated ubiquitin to substrates, thereby dictating ubiquitin chain topology and substrate fate [14]. Notably, UBE2Q2 has been frequently reported to be overexpressed in multiple malignancies, including gastric, liver, and head and neck cancers [15, 16, 17]. However, its expression pattern in IVDD remains unexplored.

Through integrative multi‐omics analysis combined with experimental validation using degenerative nucleus pulposus tissues from both humans and rats, as well as a rat nucleus pulposus cell degeneration model, we demonstrated for the first time that UBE2Q2 is significantly upregulated in IVDD samples and is predominantly enriched in nucleus pulposus cells. These findings suggest that UBE2Q2 may serve as a potential biomarker for IVDD and provide a new molecular target for its treatment, thereby providing a basis for future mechanistic and translational studies.

## 2 Materials and Methods

### 2.1 Data Acquisition and Pre‐Processing

Gene expression data from GSE70362 and GSE23130 were retrieved from the GEO database. Both datasets contain transcriptomic profiles of nucleus pulposus tissue from patients with intervertebral disc degeneration. GSE70362 (platform: GPL17810) originally comprised 48 samples; after exclusion of 24 annulus fibrosus samples, 16 non‐degenerated and 8 degenerated nucleus pulposus samples were retained. GSE23130 (platform: GPL1352) included 17 non‐degenerated and 6 degenerated nucleus pulposus samples. Platform annotation files were obtained, and probes were annotated for each dataset. Expression profiles within each dataset were then normalized using the normalizeBetweenArrays function in the limma package. The datasets were subsequently merged, and batch effects were corrected using the ComBat function in the sva package.

### 2.2 Identification of Differentially Expressed Genes (DEGs)

Differential expression analysis was conducted using the limma package to identify DEGs. DEGs were screened based on |log_2_FC| > 0 and *p* < 0.05 and categorized as upregulated, downregulated, or non‐significant. Volcano plots were generated with the ggplot2 package to visualize the DEGs.

### 2.3 Acquisition of Ubiquitination‐Related Genes

Ubiquitination‐related genes were obtained from GeneCards, with only those having a relevance score > 7 retained as UbiDEGs.

### 2.4 Functional Enrichment Analysis

The DEGs and ubiquitination‐related genes were uploaded to an online bioinformatics platform(https://www.bioinformatics.com.cn/) [18] to generate Venn diagrams, overlapping genes were designated as IVDD‐associated ubiquitination‐related DEGs (UbiDEGs). UbiDEGs were then submitted to Sangerbox 3.0(http://www.sangerbox.com/) [19] for GO and KEGG enrichment analyses (FDR < 0.05, *p* < 0.05). The results were visualized using the gseaplot2 function from the enrichplot package.

### 2.5 Weighted Gene co‐Expression Network Analysis

WGCNA was conducted on the merged dataset using the WGCNA package in R. The gene expression matrix was loaded, and the pickSoftThreshold function was applied to select the optimal soft‐thresholding power for scale‐free network construction. Scale‐free topology was confirmed for the resulting network. An adjacency matrix was created with the adjacency function and transformed into a topological overlap matrix (TOM) to quantify intramodular connectivity. A dissimilarity matrix (1—TOM) was computed and subjected to hierarchical clustering to generate a gene dendrogram. Modules were identified via dynamic tree cutting using cutreeDynamic (minModuleSize = 30) and visualized. Closely related modules (mergeCutHeight = 0.5) were merged to simplify the network, and the final dendrogram was produced. Module–trait correlations were assessed to identify the module exhibiting the strongest association with IVDD, along with its member genes.

### 2.6 Single‐Cell RNA Sequencing Analysis and Data Sources and Preprocessing

Single‐cell RNA‐seq data were obtained from the public dataset GSE251686, comprising single‐cell transcriptomes of human degenerated and non‐degenerated nucleus pulposus tissues. Raw data were processed with Seurat (v4.3.0). Low‐quality cells were filtered (number of genes < 200 or > 6000; mitochondrial content > 20%). After retaining high‐quality cells, data were normalized using NormalizeData (LogNormalize method).

### 2.7 Clustering and Cell Annotation

The top 2000 highly variable features were selected with FindVariableFeatures, followed by PCA (dims = 1–30). Clustering was performed using FindNeighbors and FindClusters (resolution = 0.8), with visualization via RunUMAP. Cell types were annotated according to established marker genes: nucleus pulposus cells (NP cells; high COL2A1, ACAN), annulus fibrosus cells (AF cells; high COL1A1), chondrocyte‐like NP cells (CHI3L1, COL10A1), and immune/endothelial cells.

### 2.8 Differential Expression and Trajectory Analysis

Within NP cell subpopulations, DEGs between IVDD and control groups were identified using FindMarkers (Wilcoxon test; min.pct = 0.25, logfc.threshold = 0.25). UBE2Q2 expression levels and cell proportion differences in degenerated NP cells were visualized with VlnPlot and FeaturePlot.

### 2.9 Establishment of Rat Intervertebral Disc Degeneration Model

Four‐week‐old male Sprague–Dawley rats were obtained from the Experimental Animal Center of Zhejiang Chinese Medical University (China). All procedures were approved by the university's Animal Ethics Committee. Rats were maintained in a specific pathogen‐free facility under a 12‐h light/dark cycle with ad libitum access to food and water. Animals were randomly allocated to experimental groups. Anaesthesia was induced by intraperitoneal pentobarbital sodium (45 mg/kg), followed by placement in the prone position. The Co6/7 disc was punctured dorsally with a 20‐gauge needle inserted centrally, advanced to the contralateral side, rotated 180°, and held for 10 s. Adjacent Co5/6 or Co7/8 discs remained intact as internal controls. Control animals underwent no puncture.

### 2.10 Quantitative Real‐Time PCR

Total RNA was extracted using an RNA isolation kit (Accurate Biology, China). RNA concentration was quantified with a NanoDrop ND‐1000 spectrophotometer. Reverse transcription was performed with the Evo M‐MLV kit (Accurate Biology, China). qPCR was conducted using SYBR Green Master Mix (Yeasen, China). For cytosolic DNA quantification, 20 ng double‐stranded DNA was analysed per reaction.

### 2.11 Western Blot

Cells were lysed in buffer (FDbio, China) containing phosphatase and protease inhibitors (Beyotime, China). Protein concentration was determined by BCA assay (Beyotime, China). Samples were mixed with loading buffer (FDbio, China) and denatured at 100°C for 7 min. Proteins were resolved by SDS‐PAGE and transferred to PVDF membranes (Millipore, USA). Membranes were blocked in 5% BSA for 1 h at room temperature, then incubated overnight at 4°C with primary antibodies (anti‐UBE2Q2 and anti‐GAPDH; Proteintech, China). After three TBST washes, membranes were incubated with HRP‐conjugated secondary antibodies (Proteintech, China) for 1 h at room temperature. Signals were detected by ECL (FDbio, China) and quantified using ImageJ.

### 2.12 Immunofluorescence

Cells were washed thrice in PBS and fixed in 4% paraformaldehyde for 15 min at room temperature. After three PBST washes, cells were permeabilized with 0.1% Triton X‐100 for 20 min and washed again. Blocking was performed with 5% BSA for 60 min. Cells were incubated overnight at 4°C with anti‐UBE2Q2 primary antibody (Proteintech, China). Following three PBST washes, Alexa Fluor 488‐ or 594‐conjugated secondary antibodies (Abcam, UK) were applied for 1 h at room temperature. Nuclei were counterstained with DAPI (Solarbio, China). Images were captured by confocal microscopy to assess protein localization and expression.

### 2.13 Immunohistochemistry

Four weeks post‐induction, rats were euthanized via carbon dioxide inhalation, and caudal discs were subsequently harvested and fixed in 4% paraformaldehyde. Tissues were decalcified in 15% EDTA for ≥ 4 weeks, paraffin‐embedded, and sectioned at 5 μm. Sections were deparaffinized in xylene and rehydrated through graded ethanol (100%–80%), followed by antigen retrieval in citrate buffer. After blocking with 5% BSA, sections were incubated overnight at 4°C with primary antibody. HRP‐ or fluorescence‐conjugated secondary antibody was applied at 37°C for 1 h. Sections were developed with DAB, counterstained with haematoxylin, mounted, and examined via whole‐slide scanning.

### 2.14 Statistical Analysis

All experiments were repeated independently at least three times. Data from ≥ 3 samples are expressed as mean ± standard deviation. Analyses were performed using GraphPad Prism 10 (GraphPad Software). Inter‐group comparisons used unpaired two‐tailed Student's *t*‐tests. Statistical significance was set at *p* < 0.05.

## 3 Result

**FIGURE 1:**
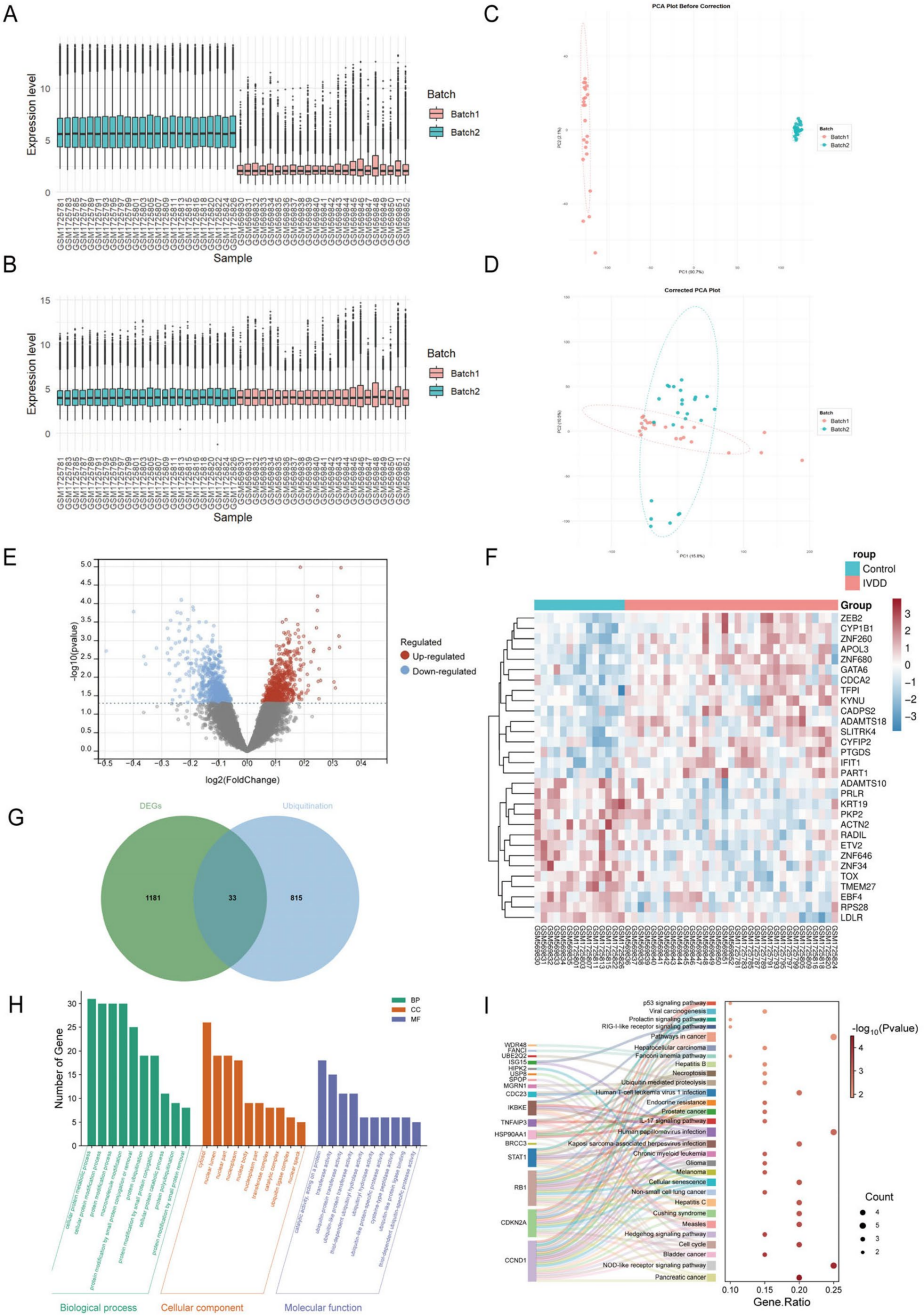
Integration of GSE Datasets, Identification of Differentially Expressed Genes, and Enrichment Analysis of Ubiquitination‐Associated Genes. (A) Boxplot of gene expression before batch effect removal. (B) Boxplot of gene expression after batch effect removal. (C) PCA plot of gene expression before batch effect removal. (D) PCA plot of gene expression after batch effect removal. (E) Volcano plot of DEGs: X‐axis, log_2_FoldChange; y‐axis, −log_10_(*p*‐value). Red, grey, and blue dots represent upregulated, non‐significant, and downregulated differentially expressed genes, respectively. (F) Heatmap displaying the top 30 upregulated and top 30 downregulated DEGs. Red and blue indicate upregulated and downregulated genes, respectively. (G) Venn diagram showing the intersection between differentially expressed genes and ubiquitination‐related genes. (H) Bar plot of GO enrichment analysis for the intersecting genes. (I) Bubble plot of KEGG pathway enrichment analysis for the intersecting genes [20, 21, 22].

Two IVDD‐related mRNA transcriptome datasets (GSE70362 and GSE23130) were retrieved from the Gene Expression Omnibus database. Following dataset integration, batch effects were corrected using ComBat (Figure 1A–D). In total, 1214 DEGs were identified, including 612 upregulated and 602 downregulated genes. Results were visualized using a volcano plot and heatmaps showing the top 30 up‐ and downregulated DEGs (Figure 1E,F). Next, 848 ubiquitination‐related genes obtained from a specialized database were deduplicated and intersected with the DEGs, resulting in 33 UbiDEGs (Figure 1G). The identified UbiDEGs underwent KEGG pathway and Gene Ontology (GO) enrichment analyses. Enrichment analysis yielded 234 significant terms: 191 biological processes (BP), 13 cellular components (CC), and 30 molecular functions (MF). The top 10 enriched terms per GO category were visualized (Figure 1H). The enriched biological processes were predominantly related to cellular protein metabolism, protein modification, and protein ubiquitination. Enriched cellular components mainly encompassed the cytoplasm, nucleus, and ubiquitin ligase complex. The enriched molecular functions primarily involved protein‐targeted catalytic activity, transferase activity, and ubiquitin‐protein transferase activity. Several key pathways were implicated in IVDD progression, including NOD‐like receptor, Hedgehog, ubiquitin‐mediated proteolysis, RIG‐I‐like receptor, cellular senescence, p53, and IL‐17 signalling pathways (Figure 1I).

**FIGURE 2:**
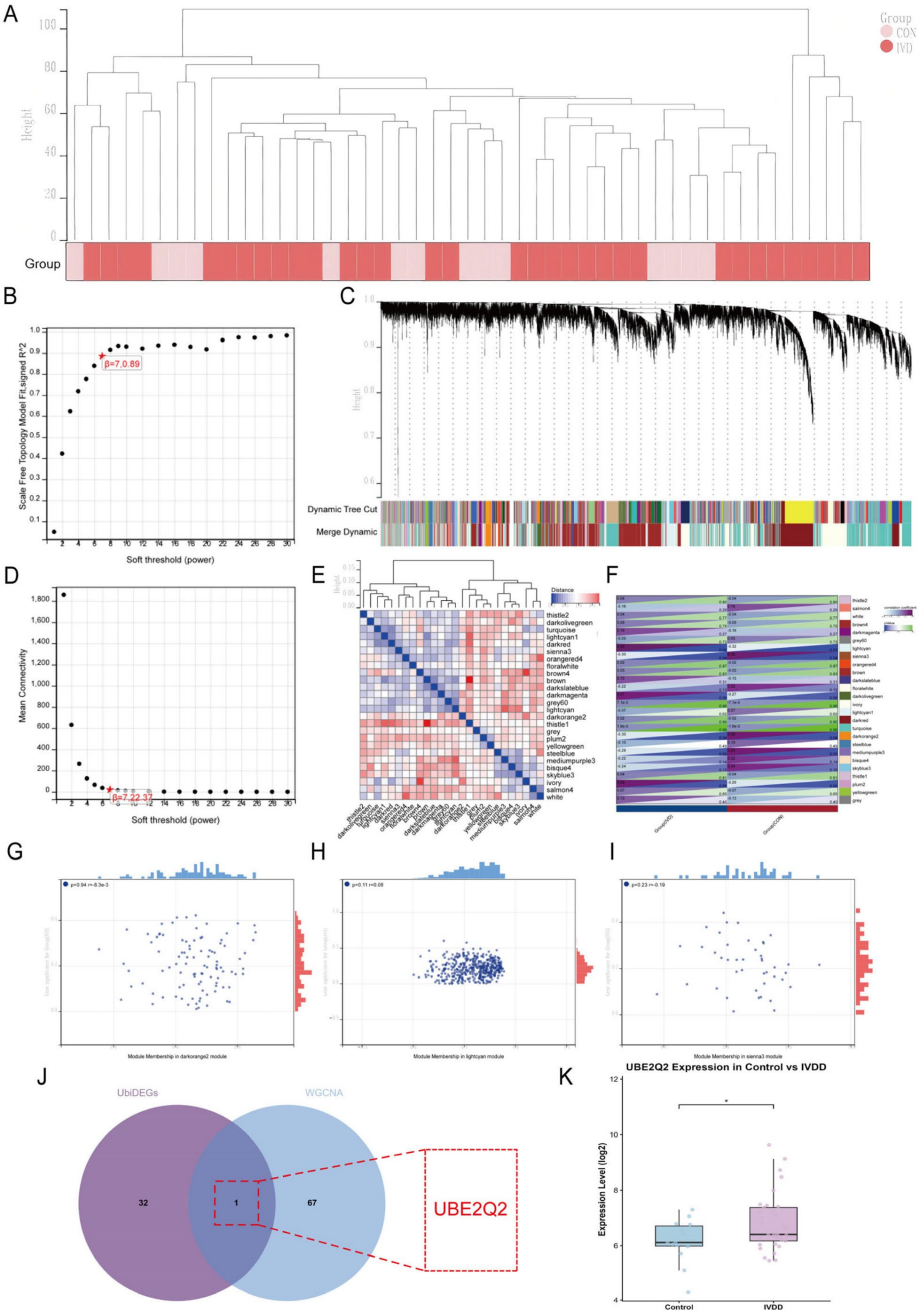
WGCNA Identification of Key Modules Related to Intervertebral Disc Degeneration and Screening of UBE2Q2. (A) Sample clustering dendrogram (cut height = 100). (B, D) Scale‐free topology model fit indices as a function of soft‐thresholding power. (C) Dynamic tree cutting dendrogram with module merging (similarity threshold = 0.5). (E) Module eigengene adjacency heatmap. (F) Module–trait correlation heatmap. (G–I) Module membership vs. gene significance scatter plots for the darkorange2, lightcyan, and sienna3 modules in relation to IVDD. (J) Venn diagram showing the overlap between UbiDEGs and genes from the key WGCNA modules. (K) Differential expression of UBE2Q2 between the control and IVDD groups.

Weighted gene co‐expression network analysis was next performed to identify gene modules associated with intervertebral disc degeneration. The sample clustering dendrogram (Figure 2A) was cut at a height of 100, retaining 47 samples for downstream analysis. The optimal soft‐thresholding power was determined to be 7 (Figure 2B,D). Dynamic tree cutting and module merging (Figure 2C) resulted in the combination of modules with similarity > 0.5, producing 26 final modules. The module eigengene adjacency heatmap (Figure 2E) showed correlations between module eigengenes, with red and blue denoting positive and negative correlations, respectively. The module–trait correlation heatmap (Figure 2F) demonstrated significant associations between the darkorange2, lightcyan, and sienna3 modules and IVDD (*p* < 0.05). These associations were further illustrated by module membership scatter plots (Figure 2G–I), leading to the selection of 68 hub genes for detailed analysis. UBE2Q2 was subsequently identified as the overlapping gene with UbiDEGs (Figure 2J). Finally, differential expression analysis confirmed significantly higher UBE2Q2 expression in the IVDD group compared with controls (Figure 2K).

**FIGURE 3:**
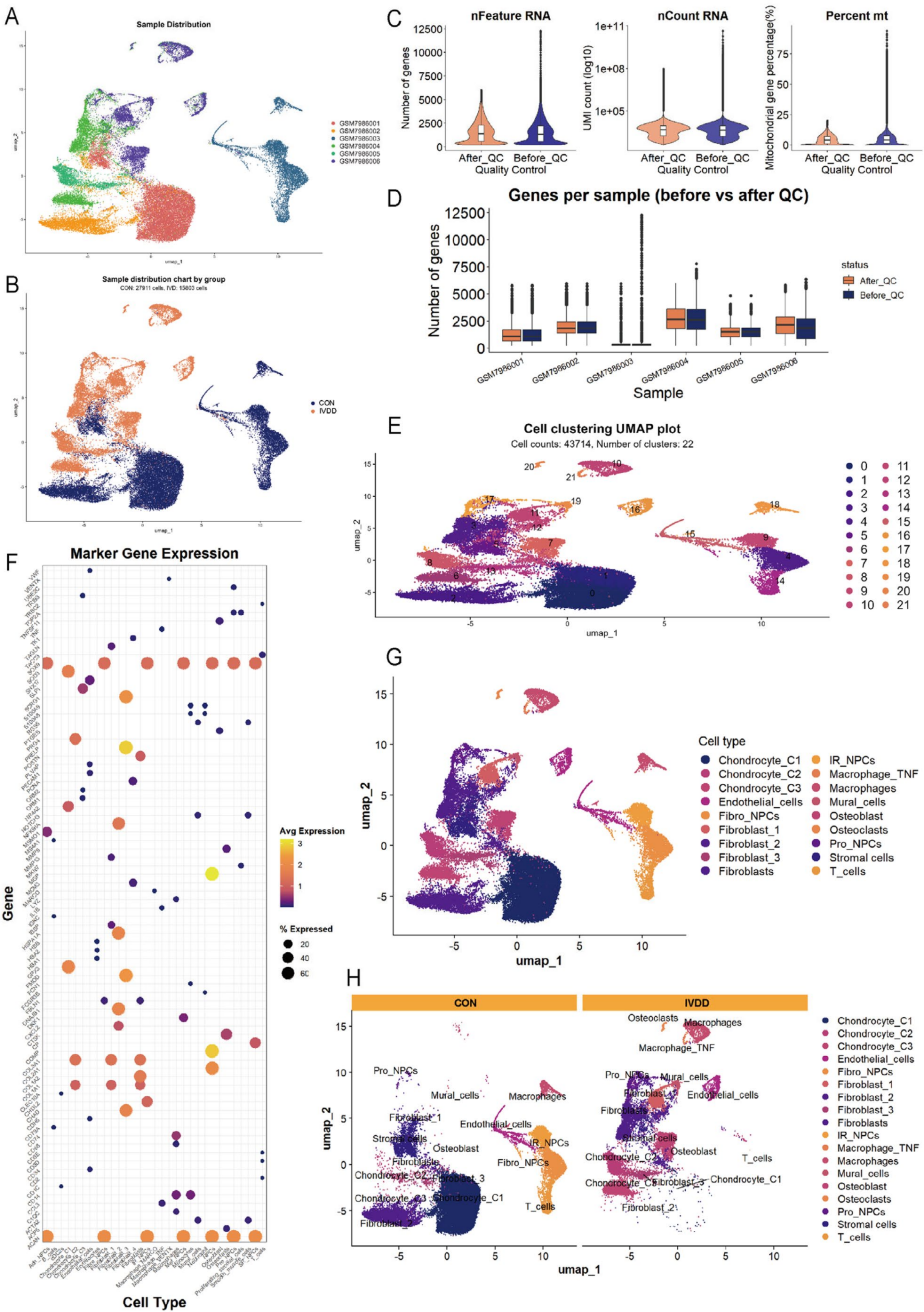
Identification and Cell Type Annotation of Intervertebral Disc Cell Clusters by Single‐Cell RNA Sequencing. (A, B) Schematic diagram showing the source distribution of included single‐cell transcriptomic samples. (C, D): Quality control of single‐cell transcriptomic samples. (E) Unsupervised clustering analysis of single cells. (F) Differentially expressed genes across cell clusters. (G) Distribution of identified cell types. (H) Abundance statistics of identified cell types.

Single‐cell RNA sequencing analysis was subsequently performed on dataset GSE251686. The dataset comprised six human intervertebral disc samples, stratified into normal (*n* = 3, dark purple) and degenerated (*n* = 3, orange) groups according to pathological status (Figure 3A,B). Following quality control, cells were retained if they had 200–6000 detected genes (nFeature_RNA)—to exclude low‐quality cells, debris, doublets, or multiplets—and mitochondrial content < 20% (percent.mt)—to remove stressed or apoptotic cells (Figure 3C,D). High‐quality cells were clustered into 22 transcriptionally distinct clusters. Cells were visualized in two‐dimensional UMAP space, with each point representing one cell and colour denoting cluster identity (Figure 3E). Differential expression analysis was conducted across all 22 clusters to identify cluster‐specific marker genes and support cell‐type annotation. The FindAllMarkers function (Wilcoxon rank‐sum test) was used to identify significantly upregulated genes per cluster (adjusted *p* < 0.05, avg. log_2_FC > 0); the top 25 markers per cluster were selected. The four most discriminative marker genes per cluster are shown (Figure 3F). In the dot plot, rows represent genes, columns represent clusters, dot size indicates the fraction of expressing cells, and colour intensity reflects average normalized expression (Figure 3F). The 22 clusters were systematically annotated, resulting in 18 well‐defined cell types (Figure 3G). Absolute cell counts were quantified for each of the 18 identified cell types to assess their relative abundance in intervertebral disc tissue. Absolute cell numbers per cell type are presented (Figure 3H). Chondrocytes, fibroblasts, and nucleus pulposus cells were the predominant cell populations in intervertebral disc tissue (Figure 3H).

**FIGURE 4:**
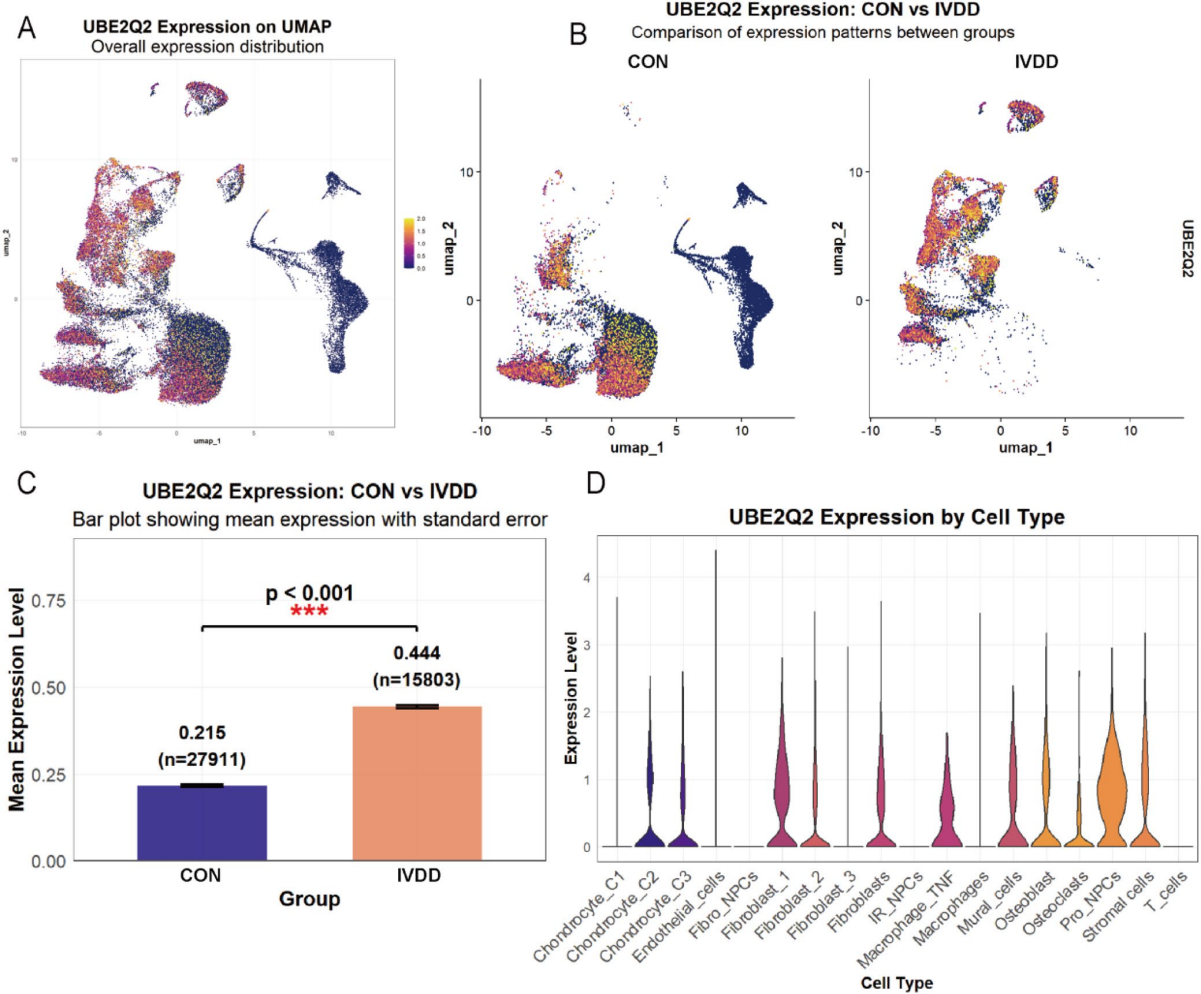
Cell‐Type‐Specific Expression Profile of UBE2Q2 in the Intervertebral Disc Single‐Cell Transcriptome. Expression profile of UBE2Q2 in the intervertebral disc single‐cell transcriptome (Fig. A–B). Differential expression analysis of UBE2Q2 during intervertebral disc degeneration (Fig. C). Expression distribution characteristics of UBE2Q2 across specific cell types (Fig. D).

To further elucidate the cell‐type‐specific expression of UBE2Q2 in intervertebral disc degeneration, we analysed the publicly available single‐cell RNA‐seq dataset from human intervertebral disc tissue (GSE251686). UBE2Q2 showed basal expression in nucleus pulposus tissue (Figure 4A,B). Differential expression analysis between IVDD and control samples showed significantly higher UBE2Q2 expression in the degenerated group (Figure 4C). UBE2Q2 was predominantly expressed in nucleus pulposus and annulus fibrosus cells, with the highest proportion of expressing cells found in NP cell subpopulations (Figure 4D). These results suggest that UBE2Q2 primarily functions in nucleus pulposus cells and is involved in their pathological changes during IVDD progression.

**FIGURE 5:**
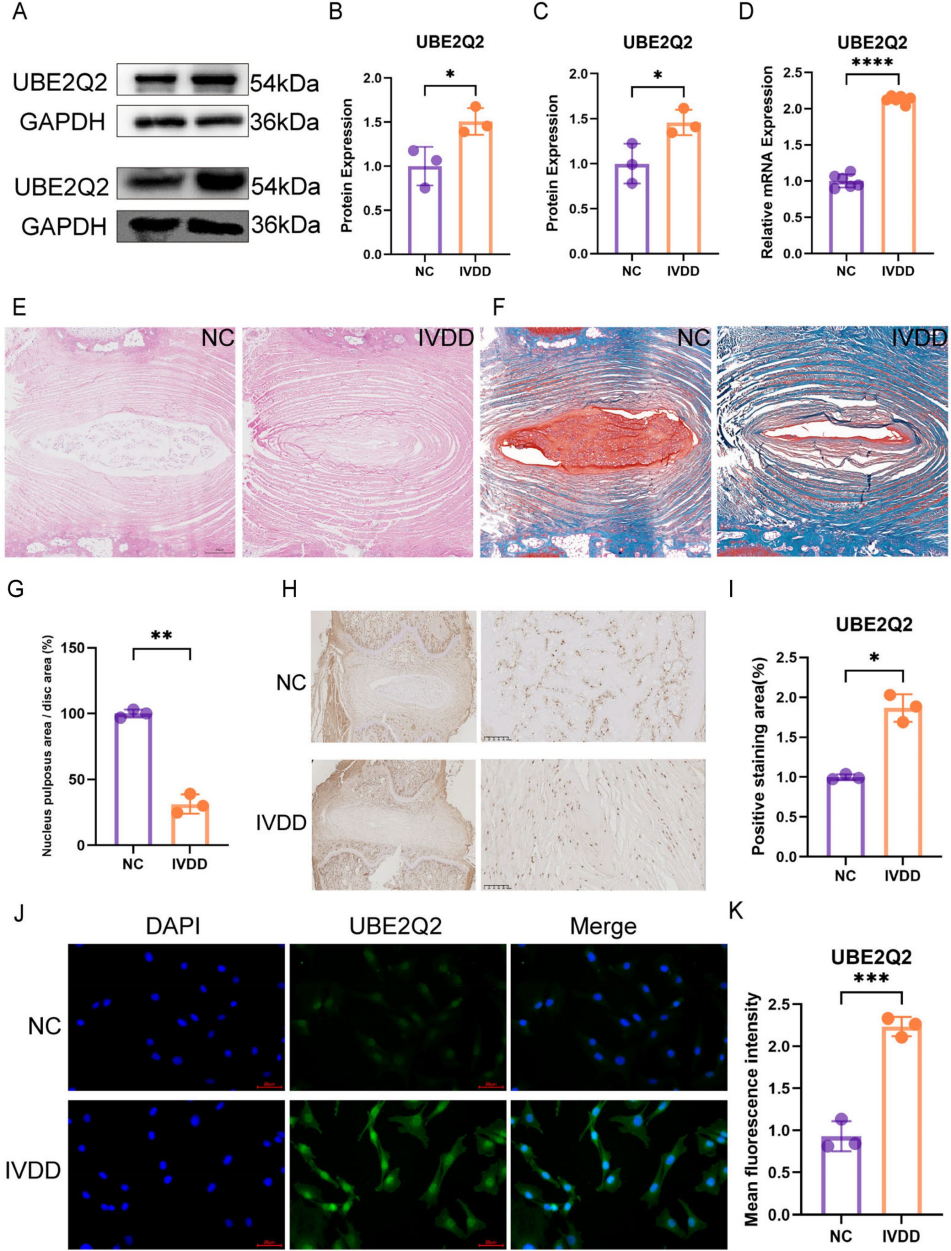
Upregulation of UBE2Q2 mRNA and Protein Expression in Degenerated Nucleus Pulposus Tissue and Cells. (A) Representative Western blot images showing UBE2Q2 and GAPDH in primary rat nucleus pulposus cells (top panel) and in human normal control and degenerated nucleus pulposus tissue (bottom panel). (B) Quantitative analysis of UBE2Q2 protein expression in primary nucleus pulposus cells (*n* = 3; *p < 0.05). (C) Quantitative analysis of UBE2Q2 protein expression in human nucleus pulposus tissue (*n* = 3; *p < 0.05). (D) qRT‐PCR analysis of relative UBE2Q2 mRNA expression in human nucleus pulposus tissue (*n* = 6; ****p < 0.0001). (E) H&E staining of intervertebral discs. Representative images of Haematoxylin and Eosin staining in the NC and IVDD groups. (F) Safranin O staining of intervertebral discs. Representative images of Safranin O staining showing proteoglycan distribution in the NC and IVDD groups. (G) Quantitative analysis of nucleus pulposus area. Box plot showing the comparison of nucleus pulposus area between the NC and IVDD groups (*n* = 3, **p < 0.01). (H) Representative immunohistochemical staining of UBE2Q2 in rat nucleus pulposus tissue: Control group (NC, top) and IVDD group (bottom). Scale bar: 100 μm. (I) Semi‐quantitative analysis of the percentage of UBE2Q2‐positive staining area in rat nucleus pulposus tissue (*n* = 3; *p < 0.05). (J) Representative immunofluorescence staining of UBE2Q2 (green) and DAPI (blue) in primary nucleus pulposus cells: Control group (NC, top) and IVDD group (bottom). Merged images are shown on the right. Scale bar: 20 μm. (K) Quantitative analysis of the percentage of UBE2Q2‐positive staining area in primary nucleus pulposus cells (*n* = 3; ***p < 0.001, independent samples). Data are presented as mean ± standard deviation.

To validate the bioinformatics and single‐cell findings, UBE2Q2 expression was assessed in human clinical nucleus pulposus tissue, primary rat nucleus pulposus cells, and a rat IVDD model. Western blot analysis revealed significantly elevated UBE2Q2 protein levels in human degenerated nucleus pulposus tissue compared with normal controls (Figure 5A,C). Similarly, UBE2Q2 protein was significantly upregulated in primary rat nucleus pulposus cells of the IVDD group (Figure 5A,B). qRT‐PCR confirmed markedly higher UBE2Q2 mRNA levels in IVDD nucleus pulposus tissue than in controls (*p* < 0.0001; Figure 5D). To confirm successful establishment of the rat IVDD model, this study conducted histological examination of disc tissues from the normal control (NC) and IVDD groups using haematoxylin–eosin (H&E) and Safranin O (SO) staining. H&E staining revealed an intact annulus fibrosus (AF) structure and clear nucleus pulposus (NP) boundaries in the NC group. In contrast, the IVDD group exhibited typical degenerative changes, including lamellar disruption and delamination of the annulus fibrosus, decreased nucleus pulposus area, and fibrotic tissue infiltration into the NP region (Figure 5E,G). Safranin O staining further demonstrated significant proteoglycan loss in the IVDD group, evidenced by markedly reduced red staining intensity in the nucleus pulposus and inner annulus fibrosus, whereas the NC group showed strong and uniform Safranin O‐positive matrix. These morphological and histochemical alterations were consistent with the characteristic features of intervertebral disc degeneration (Figure 5F). These observations were further validated in the rat IVDD model by immunohistochemistry. In control nucleus pulposus tissue, UBE2Q2 showed weak or negative, predominantly cytoplasmic staining. In contrast, IVDD tissue exhibited abundant brownish‐yellow positive granules with substantially increased staining intensity and positive area (Figure 5H). Semi‐quantitative analysis demonstrated a significantly greater percentage of positive staining area in the IVDD group than in controls (*p* < 0.05; Figure 5I). Immunofluorescence staining in primary nucleus pulposus cells further confirmed UBE2Q2 protein upregulation. Control cells exhibited weak green UBE2Q2 fluorescence, whereas IVDD cells displayed significantly stronger signals (Figure 5J). Quantitative analysis indicated that the UBE2Q2‐positive area percentage in the IVDD group was approximately twice that of controls (*p* < 0.05; Figure 5K).

Collectively, Western blot, qRT‐PCR, IHC, and IF results consistently showed significant UBE2Q2 upregulation at both mRNA and protein levels in IVDD nucleus pulposus tissue and cells, indicating a potential key role in intervertebral disc degeneration progression.

## 4 Discussion

This study systematically evaluated the expression profile of the ubiquitin‐conjugating enzyme UBE2Q2 in intervertebral disc degeneration using integrative multi‐omics analysis combined with single‐cell transcriptomic data and multi‐level experimental validation. The results consistently demonstrated that UBE2Q2 was significantly upregulated in degenerated nucleus pulposus tissues and cells. It was predominantly enriched in the nucleus pulposus cell population, suggesting a close association with the onset and progression of IVDD. These findings established a foundation for further research on UBE2Q2 as a potential molecule implicated in IVDD.

First, integrative analysis of two GEO transcriptomic datasets identified 1214 differentially expressed genes. Intersection with a set of ubiquitination‐related genes yielded 33 IVDD‐associated ubiquitin‐related DEGs. Functional enrichment analysis showed that these genes are mainly involved in protein metabolism, protein modification, and ubiquitination processes. They were also significantly enriched in the IL‐17, p53, and cellular senescence signalling pathways. These pathways have been shown to play key roles in the pathogenesis of IVDD [23, 24, 25, 26].

Weighted gene co‐expression network analysis subsequently identified co‐expression modules significantly correlated with IVDD. Further analysis identified UBE2Q2 as a hub gene shared by both key modules and the UbiDEGs set. Differential expression analysis confirmed significant upregulation of UBE2Q2 in degenerated intervertebral disc samples. Notably, single‐cell RNA sequencing further revealed the cell‐type‐specific expression profile of UBE2Q2. Results indicated that UBE2Q2 was predominantly expressed in nucleus pulposus cells and exhibited significantly higher expression in degenerated samples. Nucleus pulposus cells represent the primary cell population maintaining intervertebral disc homeostasis. Their apoptosis and senescence are widely recognized as pivotal events in IVDD pathogenesis [27, 28]. Previous research has demonstrated that TRIM family E3 ligases exert pleiotropic effects in IVDD through regulation of inflammatory signalling, apoptosis, and extracellular matrix homeostasis. Their aberrant expression is closely associated with degeneration severity [29]. Likewise, UBE2C, an E2 enzyme, regulates NP cell proliferation, inflammatory mediator release, and ECM metabolism via macrophage polarization, indicating its potential as a pro‐degenerative factor in IVDD [30]. These observations suggest that ubiquitin‐proteasome system enzymes at different hierarchical levels function as key regulatory nodes in IVDD, with E2 conjugating enzymes serving as critical determinants of ubiquitin chain type and substrate specificity [10, 31]. Our multi‐omics analyses consistently indicate elevated UBE2Q2 expression in degenerated intervertebral discs. Nonetheless, further experimental validation is required.

To validate these findings, UBE2Q2 expression was further assessed in clinical degenerated nucleus pulposus tissues, primary rat nucleus pulposus cells, and a puncture‐induced rat model of IVDD. Western blot, qRT‐PCR, immunohistochemistry, and immunofluorescence analyses consistently confirmed significant UBE2Q2 upregulation in both degenerated tissues and cells. The marked consistency across these results greatly enhances the reliability of our conclusions. Currently, UBE2Q2 function in malignancies is well documented. For example, it drives glycolysis and tumour progression in hepatocellular carcinoma through NF‐κB signalling and promotes cell survival in inflammatory microenvironments [15]. Notably, IVDD shares several key pathological features with these processes, including chronic inflammation, metabolic reprogramming, and oxidative stress. These parallels raise the possibility that UBE2Q2 may contribute to degenerative changes in NP cells, although the precise mechanisms in the context of IVDD remain to be elucidated [32].

Although this study provides substantial evidence, several limitations should be acknowledged. First, the datasets included a limited number of donors and may not adequately capture the genetic variability of the broader population. Second, this is a preliminary screening study. The specific substrates of UBE2Q2 and its downstream signalling pathways remain to be clearly defined. Future studies will focus on functional validation and in vivo intervention using gene knockout or overexpression models to obtain direct causal evidence for UBE2Q2. Third, age differences exist between groups in the human clinical samples. Future studies will utilize age‐matched control specimens to further validate these findings, thereby minimizing age‐related confounding factors to the greatest extent possible. These approaches will help elucidate its precise role in intervertebral disc degeneration and assess its potential as a therapeutic target.

## 5 Conclusion

This study is the first to identify UBE2Q2 as a significantly upregulated ubiquitin‐conjugating enzyme in IVDD, which is primarily expressed in nucleus pulposus cells. Its consistent upregulation across transcriptomic, single‐cell, and experimental datasets suggests that UBE2Q2 may serve as a potential biomarker and a contributing factor in the degenerative process. These findings lay the foundation for future functional studies.

## Author Contributions

**Wenqiang Cheng:** conceptualization, validation, writing – original draft. **Fangfang Liu:** methodology, validation. **Jianye Yang:** methodology, validation. **Meijun Chen:** data curation, investigation. **Jintao Hu:** supervision, writing – review and editing. **Weihui Qi:** funding acquisition, writing – review and editing, supervision. **Dong Wang:** writing – review and editing, funding acquisition, supervision. **Hao Pan:** funding acquisition, writing – review and editing.

## Funding

This work was supported by the National Natural Science Foundation of China (No: 82405429); The Medical and Health Science and Technology Program of Hangzhou, China (No: A20230195); The Key Research and Development Program of Agriculture and Social Development of Hangzhou Science and Technology Bureau, China (No: 20241203A15); Key Discipline of Traditional Chinese Medicine in Zhejiang Province (2024‐XK‐57) and The Construction Fund of Key Medical Discipline of Hangzhou (2025HZZD16).

## Disclosure

During the preparation of this manuscript, ChatGPT was used solely for language translation and polishing of selected text sections. All artificial intelligence‐generated content was carefully reviewed, edited, and verified for accuracy by the authors. No artificial intelligence tools were used to generate original research ideas, conduct data analyses, provide scientific interpretations, or produce the core content of the manuscript.

## Ethics Statement

The study protocol involving human participants was approved by the Ethics Committee of Hangzhou Hospital of Traditional Chinese Medicine (Approval number: 2022KY039). Normal nucleus pulposus samples were obtained from patients aged 27–31 years who had sustained burst fractures. These non‐degenerated intervertebral discs were classified as Pfirrmann grade I based on T2‐weighted MRI and the Pfirrmann grading system. Degenerated human NP tissue samples were collected from patients aged 56–62 years with lumbar disc herniation, with the corresponding discs graded as Pfirrmann grade IV. None of the enrolled patients had a history of cardiovascular or cerebrovascular disease, cancer, infection, immune disorders, endocrine disease, or organ dysfunction. Three pairs of samples (three normal and three degenerated) were used for Western blot analysis. All participants provided written informed consent for the use of their tissue samples in research. All animal experiments, including animal sourcing, surgery, and tissue collection, were approved by the Animal Ethics Committee of Zhejiang Chinese Medical University (Approval number: 20250331–08). All methods were carried out in accordance with relevant guidelines and regulations.

## Conflicts of Interest

The authors declare no conflicts of interest.

## Data Availability

The data that support the findings of this study are available from the corresponding author upon reasonable request.
